# Use of Radiation Therapy in Metastatic Nasopharyngeal Cancer Improves Survival: A SEER Analysis

**DOI:** 10.1038/s41598-017-00655-1

**Published:** 2017-04-07

**Authors:** Jiyi Hu, Lin Kong, Jing Gao, Weixu Hu, Xiyin Guan, Jiade J. Lu

**Affiliations:** 1 Department of Radiation Oncology, Shanghai Proton and Heavy Ion Center, Shanghai, 201321 China; 2 Department of Radiation Oncology, Shanghai Proton and Heavy Ion Center, Fudan University Cancer Hospital, Shanghai, 201321 China

## Abstract

Limited data indicated radiotherapy might provide survival benefits to patients with distantly metastatic nasopharyngeal carcinoma (mNPC). We used the Surveillance Epidemiology and End Results database to examine the role of radiotherapy in the treatment of mNPC. Patients with mNPC at presentation diagnosed between 1988 and 2012 were enrolled. The outcome of interest included overall survival (OS) and cancer-specific survival (CSS). A total of 679 patients with a median follow-up of 13 months were identified. Four hundred forty-eight patients received radiotherapy and 231 did not. Radiotherapy was associated with significantly improved OS and CSS in both univariate and multivariate analyses. Weighted Cox regression by inverse probability of treatment weighting (IPTW) using propensity score (PS) showed a 50% reduced risk of mortality in patients who received radiotherapy with regards to both OS (HR: 0.50, 95% CI: 0.41–0.60, p < 0.001) and CSS (HR: 0.50, 95% CI: 0.40–0.61, p < 0.001), respectively. Further, patients with a younger age (<65 year-old), diagnosed after 2003, with non-keratinizing carcinoma or undifferentiated carcinoma, and who received surgery had better outcomes for both OS and CSS. Local radiotherapy was associated with improved survival in patients with mNPC. Our findings warrant prospective investigation in clinical trials.

## Introduction

Nasopharyngeal cancer (NPC) is highly sensitive to both radiation therapy and chemotherapy. Radiation alone is the treatment of choice for early stage NPC, and concurrent chemoradiation is the current standard for locoregionally advanced disease^1^. The prevailing use of intensity-modulated radiation therapy (IMRT) with or without chemotherapy has significantly improved outcome after definitive treatment for NPC. However, approximately 10% of patients present with metastatic NPC (mNPC) at initial diagnosis, and an additional 10% of patients develop metastatic failure after definitive treatment^2^. Metastatic NPC (mNPC) is an incurable condition. Based on the NCCN guideline for the management of head and neck cancers^3^, cisplatin-based chemotherapy is considered its initial management choice for mNPC, and radiation therapy for the primary lesion is only recommended for patients who achieved complete response after systemic chemotherapy.

The addition of local therapy such as radiation to systemic chemotherapy for metastatic cancer has been practiced for a number of diseases with success in improving treatment outcome including survival. Despite data demonstrating improved survival with tumor burden reduction using radiotherapy in metastatic foci^4, 5^ and primary tumor^6–8^ in other malignancies, the recommended utilization of active radiotherapy or chemoradiation for mNPC patients by NCCN is not supported by any large-scale observational or prospective studies. Potential benefit of radiotherapy was only suggested by case reports and retrospective series with limited sample size^9–11^. Therefore, we conducted a study using the Surveillance, Epidemiology, and End Results (SEER) database to determine whether NPC patients diagnosed with distant metastasis who received radiation therapy had an improved survival compared with patients who did not.

## Materials and Methods

### Patients

The SEER registry, which captures 28% of the American population, contains information on patient demographics, tumor characteristics, and choice of primary treatment modality^12^. We queried the SEER Registry using SEERSTAT version 8.2.1 to identify patients with metastatic NPC diagnosed from 1988 through 2012.

Patients with nasopharyngeal carcinoma were identified by International Classification of Disease-O-3 (ICD-O-3) site codes C110-C119 and histologic codes 8010, 8020, 8070–8073, 8021 and 8082. Metastatic cases were defined as extent of disease (EOD-10) code 85 (for cases before 2004) and Collaborative staging metastasis at diagnosis (CS Mets at Dx) code 10, 40, 50 and 60 (for cases diagnosed at 2004 and after). Histology of NPC was classified into squamous cell carcinoma (ICD-O-3 codes 8070, 8071), non-keratinizing carcinoma (ICD-O-3 codes 8072, 8073), undifferentiated carcinomas (ICD-O-3 codes 8020, 8021 and 8082) and not otherwise specified group (ICD-O-3 code 8010). Only patients treated with beam radiation (with or without other type of radiotherapy) were included. Cases with missing data on the status of radiotherapy were excluded.

### Statistical Analysis

The difference in baseline demographic characteristics between patients treated with and without radiotherapy were compared by Pearson’s chi-squared test or Fisher’s exact test for categorical variables. Outcomes of interest included overall survival (OS) and nasopharyngeal cancer-specific survival (CSS) based on complete-case analyses. For univariate analyses, Kaplan-Meier method with log-rank test and univariate Cox proportional hazards (PH) model were used. Age, gender, race, histology, year of diagnosis, status of surgery and radiotherapy were included in the multivariate analysis by Cox PH model.

Multiple imputation by MICE was used for missing values regarding to race, grade, histology and surgery^13^. All available covariates, the log form of survival time, event indicator, as well as cumulative hazard *H*_*0*_(*T*) were used as predictors for multiple imputation. Five imputed datasets were generated. Univariate and multivariate analyses by Cox regression were performed on each imputed dataset. Results were then pooled by Rubin’s Rules.

In order to balance the difference in the baseline characteristics between patients treated with and without radiotherapy, inverse probability of treatment weighting (IPTW) using propensity score was applied^14–16^. Logistic regression was used to generate the propensity scores of the baseline characteristics. The IPTW was defined as (Z/e) + [(1 − Z)/(1 − e)] (Z = 1 denotes radiotherapy received while Z = 0 denotes radiotherapy not received; e denotes propensity score)^17^. The IPTWs were then stabilized to avoid extreme weights that may result in unreliable outcome^18^. Multivariate analyses were performed by Cox PH model with the IPTWs incorporated. Standardized mean difference (SMD) was used to evaluate the balance of baseline characteristics before and after IPTW using PS.

P values < 0.05 (2-sided) were considered statistically significant. All statistical analyses were performed using R environment for statistical computing and graphics (version. 3.2.3).

## Results

### Demographic characteristics

A total of 679 cases with metastatic NPC diagnosed between 1988 and 2012 were identified. Among those, 448 patients (66.0%) received radiotherapy while 231 patients (34.0%) did not.

The median follow-up was 13 months, and mortality occurred in 513 (75.6%) patients. All baseline characteristics were summarized in Table 1. Patients less than 65 years old were more likely to receive radiotherapy (64.1% vs. 74.3%, p = 0.007). While other characteristics were similar in groups with and without radiotherapy.

**Table 1:** Baseline characteristics of the study population.

Characteristics	No Radiotherapy (n (n%))	Radiotherapy (n (n%))	P
Total (N = 679)	231	448	
Age (year)			
<65	148 (64.1)	333 (74.3)	0.007
≥65	83 (35.9)	115 (25.7)	
Gender			
Male	174 (75.3)	335 (74.8)	0.950
Female	57 (24.7)	113 (25.2)	
Race			
White	115 (49.8)	191 (42.6)	0.278*
Black	30 (13.0)	74 (16.5)	
Other	85 (36.8)	181 (40.4)	
Unknown	1 (0.4)	2 (0.5)	
Year of diagnosis			
1988–2003	82 (34.5)	177 (39.5)	0.349
2004–2012	149 (65.5)	271 (60.5)	
Grade			
Grade I	4 (1.8)	6 (1.3)	0.065*
Grade II	21 (9.1)	34 (7.6)	
Grade III	77 (33.3)	174 (38.8)	
Grade IV	50 (21.6)	123 (27.5)	
Unknown	79 (34.2)	111 (24.8)	
Histology			
Squamous cell carcinoma	105 (45.5%)	188 (42.0%)	0.315
Non-keratinizing carcinoma	30 (13.0%)	82 (18.3%)	
Undifferentiated carcinoma	40 (17.3%)	81 (18.1%)	
Unknown	56 (24.2%)	97 (21.6%)	
Surgery			
No surgery	209 (90.5)	392 (87.5)	0.318*
Surgery	22 (9.5)	52 (11.6)	
Unknown	0	4 (0.9)	

^*^Fisher’s exact test was used.

### Survival analyses

**Figure 1:**
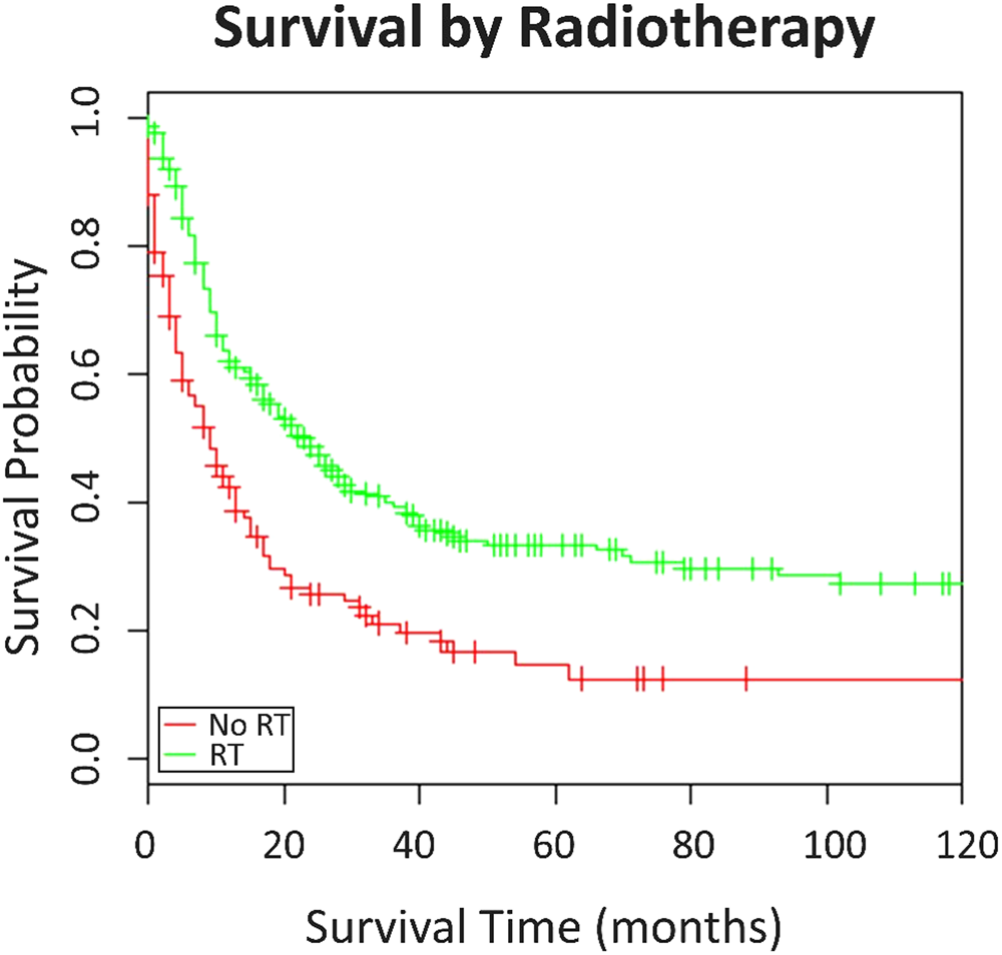
Kaplan-Meier survival curve for radiotherapy.

The results of univariate analyses by Cox regression were detailed in Table 2. Radiotherapy was associated with significantly improved overall survival (HR: 0.50, 95% CI: 0.41–0.60, p < 0.001) and cancer-specific survival (HR: 0.50, 95% CI: 0.41–0.61, p < 0.001). In addition, younger age (<65 years), disease diagnosed after 2004, races other than black and white, patients with non-keratinizing or undifferentiated carcinoma and patients who received surgery were associated with significantly improved OS and CSS. Female patients had a better CSS than males. Races other than white and black showed an improved OS. Additionally, patients with tumor grade IV had improved OS. KM survival curve for radiotherapy was shown in Fig. 1.

**Table 2:** Univariate analyses of OS and CSS.

Covariable	OS		CSS	
	HR (95%CI)	P	HR (95%CI)	P
Age (<65 as ref.)				
≥65	2.07 (1.72–2.50)	<0.001	2.01 (1.61–2.50)	<0.001
Gender (male as ref.)				
Female	0.86 (0.70–1.05)	0.146	0.79 (0.62–0.999)	0.049
Race (whites as ref.)				
Black	0.90 (0.70–1.16)	0.411	0.77 (0.57–1.04)	0.086
Other	0.81 (0.67–0.98)	0.033	0.83 (0.67–1.03)	0.098
Year of diagnosis (1988–2003 as ref.)				
2004–2012	0.74 (0.62–0.88)	<0.001	0.75 (0.61–0.92)	0.005
Grade (grade I as ref.)				
Grade II	0.65 (0.26–1.61)	0.331	0.53 (0.17–1.64)	0.246
Grade III	0.57 (0.28–1.15)	0.114	0.49 (0.16–1.48)	0.188
Grade IV	0.46 (0.23–0.92)	0.030	0.40 (0.14–1.14)	0.082
Histology (squamous cell carcinoma as ref.)				
Non-keratinizing carcinoma/undifferentiated carcinoma	0.71 (0.58–0.86)	<0.001	0.69 (0.56–0.85)	<0.001
Surgery (no surgery as ref.)				
Surgery	0.63 (0.47–0.85)	0.002	0.61 (0.43–0.87)	0.006
Radiotherapy (no radiotherapy as ref.)				
Radiotherapy	0.50 (0.41–0.60)	<0.001	0.50 (0.41–0.61)	<0.001

Consistent to univariate analyses, multivariate analyses by Cox PH model showed radiotherapy was independently associated with a 50% lower risk of mortality in regard to both OS (HR: 0.50, 95% CI: 0.42–0.61, p < 0.001) and CSS (HR: 0.50, 95% CI: 0.41–0.62, p < 0.001). Table 3 showed the results of un-weighted multivariate analyses of OS and CSS.

**Table 3:** Unweighted multivariate analyses of OS and CSS.

Covariable	OS		CSS	
	HR (95%CI)	P	HR (95%CI)	P
Age (<65 as ref.)				
≥65	1.86 (1.53–2.26)	<0.001	1.81 (1.44–2.27)	<0.001
Gender (male as ref.)				
Female	0.83 (0.68–1.02)	0.079	0.77 (0.60–0.98)	0.034
Race (whites as ref.)				
Black	0.94 (0.73–1.22)	0.65	0.77 (0.57–1.05)	0.102
Other	0.89 (0.73–1.08)	0.23	0.88 (0.71–1.10)	0.265
Year of diagnosis (1988–2003 as ref.)				
2004–2012	0.64 (0.53–0.77)	<0.001	0.67 (0.54–0.83)	<0.001
Histology (squamous cell as ref.)				
Non-keratinizing carcinoma/undifferentiated carcinoma	0.79 (0.64–0.98)	0.029	0.77 (0.61–0.96)	0.024
Surgery (no surgery as ref.)				
Surgery	0.58 (0.43–0.78)	<0.001	0.56 (0.39–0.81)	0.002
Radiotherapy (no radiotherapy as ref.)				
Radiotherapy	0.50 (0.42–0.61)	<0.001	0.50 (0.41–0.62)	<0.001

**Figure 2:**
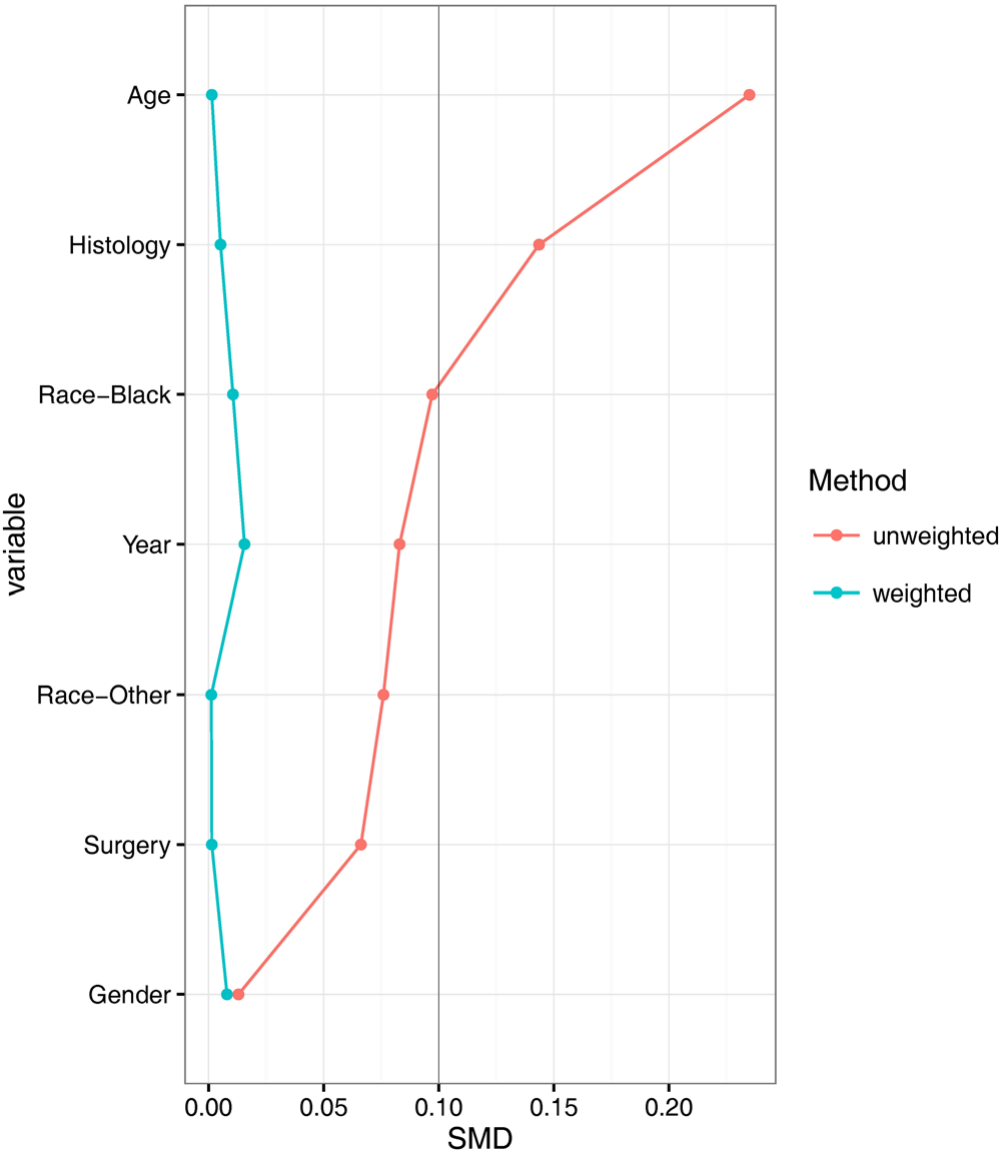
Standardized mean difference (SMD) before and after IPTW by PS.

Though chi-squared tests showed baseline characteristics were generally balanced except age, by examining the standardized mean difference (SMD) of those variables between two groups, we observed two out of six variables had a SMD larger than 10%, while the others approached 10%, indicating possible confounding (Table 4 and Fig. 2). Thus, we performed weighted Cox PH regression using IPTW derived from propensity score. After weighting, SMDs of all 6 variables were below 10%, indicating baseline characteristics were more comparable in the weighted sample.

**Table 4:** Standardized mean difference of co-variables before and after weighting by IPTW.

Covariable	Standardized mean difference (SMD)	
	Un-weighted	Weighted by IPTW
Age	0.235	0.001
Gender	0.013	0.008
Race		
Black	0.097	0.011
Other	0.076	0.001
Year of diagnosis	0.083	0.016
Histology		
Non-keratinizing carcinoma/undifferentiated carcinoma	0.144	0.005
Surgery	0.066	0.001

Table 5 shows the results of weighted multivariate analyses using IPTW by PS. Similarly, a 50% reduced risk of mortality was found for radiotherapy, for both OS (HR: 0.50, 95% CI: 0.41–0.60, p < 0.001) and CSS (HR: 0.50, 95% CI: 0.40–0.61, p < 0.001). An older age (≥65) was associated with an 91% increased risk of mortality for OS (HR: 1.91, 95% CI: 1.57–2.31, p < 0.001) and a 87% increased risk for CSS (HR: 1.87, 95% CI: 1.49–2.34, p < 0.001). Compared with earlier cases, patients diagnosed between 2004–2012 had better OS (HR: 0.63, 95% CI: 0.53–0.76, p < 0.001) and CSS (HR: 0.66, 95% CI: 0.54–0.81, p < 0.001). Surgical resection was also related to improved outcomes for both OS (HR: 0.58, 95% CI: 0.43–0.78, p < 0.001) and CSS (HR: 0.56, 95% CI: 0.39–0.80, p = 0.002). Compared to squamous cell carcinoma, patients with either non-keratinizing carcinoma or undifferentiated carcinoma had a better OS (HR: 0.77, 95% CI: 0.63–0.96, p = 0.020) and CSS (HR: 0.75, 95% CI: 0.59–0.95, p = 0.016). Female patients had a better CSS (HR: 0.78, 95% CI: 0.61–0.99, p = 0.045) but not OS (HR: 0.84, 95% CI: 0.69–1.03, p = 0.101). No significant difference was found between different races.

**Table 5:** Weighted multivariate analyses using IPTW by PS.

Co-variable	OS		CSS	
	HR (95%CI)	P	HR (95%CI)	P
Age (<65 as ref.)				
≥65	1.91 (1.57–2.31)	<0.001	1.87 (1.49–2.34)	<0.001
Gender (male as ref.)				
Female	0.84 (0.69–1.03)	0.101	0.78 (0.61–0.99)	0.045
Race (whites as ref.)				
Black	0.96 (0.74–1.23)	0.731	0.79 (0.58–1.07)	0.121
Other	0.86 (0.71–1.05)	0.143	0.86 (0.69–1.07)	0.173
Year of diagnosis (1988–2003 as ref.)				
2004–2012	0.63 (0.53–0.76)	<0.001	0.66 (0.54–0.81)	<0.001
Histology (squamous cell as ref.)				
Non-keratinizing carcinoma/undifferentiated carcinoma	0.77 (0.63–0.96)	0.020	0.75 (0.59–0.95)	0.016
Surgery (no surgery as ref.)				
Surgery	0.58 (0.43–0.78)	<0.001	0.56 (0.39–0.80)	0.002
Radiotherapy (no radiotherapy as ref.)				
Radiotherapy	0.50 (0.41–0.60)	<0.001	0.50 (0.40–0.61)	<0.001

## Discussion

NPC is the most commonly diagnosed head and neck malignancy in Southern China. Approximately 10% of patients are diagnosed with distant metastasis (i.e., Stage IVC). Although radiation therapy (or combined chemoradiation therapy) are routinely used for the treatment of non-metastatic NPC, systemic chemotherapy remains the initial management of choice for patients diagnosed with metastatic disease^3^. Despite the ability of chemotherapy to prolong survival and curtail disease-related symptoms, resistance to cytotoxic agents ultimately develops. The introduction of multi-agent regimens with the addition of newer, more novel agents such as taxotere and gemcitabine to cisplatin has shown improvement in survival. Nevertheless, the 5-year overall survival rate for patients with mNPC is only 20%^11, 19^, in stark contrast to >80% for patients diagnosed without meta stasis.

Decreasing tumor burden using radiotherapy improves survival in a number of malignancies. For example, local radiation therapy to the primary or metastatic disease improves survival in patients with metastatic breast cancer, renal cell carcinoma, prostate cancer, lung cancer, and primary brain tumor^4–8^. Prospective data do not exist regarding a survival benefit for patients with mNPC undergoing treatment of the primary tumor. However, results from a case series of 5 patients and retrospective studies with limited sample size suggested improved outcome in terms of long-term survival with radiation to the primary disease focus in NPC patients presented with metastasis at diagnosis. In their retrospective study of 125 patients diagnosed with stage IVC mNPC between 1993 and 2001, Yeh *et al*. discovered that the 1-year overall survival (OS) rates were 25%, 36%, and 48% for patients received no treatment, chemotherapy (CDDP/5-FU regimen) alone, or radiation therapy alone, respectively. The authors did not study the effect of local therapy in mNPC patients treated with systemic chemotherapy, and interestingly, patients received radiotherapy alone to the head and neck area had better OS than those received systemic chemotherapy^10^. Similar findings were reported in another retrospective study of 105 patients who were treated with chemotherapy followed by high-dose chemo-radiation. The median survival of patients in this series was 25 months, and the 2-/5-year OS rates were 50% and 17%, respectively^11^. However, the authors did not compare the OS of this group of patients with those who did not receive radiation therapy in the same institution.

Further, the results of a SEER analysis of 177 patients with mNPC diagnosed at presentation suggested that the use of radiation therapy improved OS as compared to those without^19^. The 5-year OS rates were 28% versus 3% (p < 0.0001) favoring the use of radiation therapy in mNPC patients. Furthermore, the authors also concluded that younger age and non-white race were favorable prognostic factors for mNPC. However, that report was presented in an abstract format and detailed knowledge of the analysis has never been published in a manuscript format. The number of patients included in the analysis was also limited and the treatment technology used might be obsolete as only patients diagnosed from 1988 to 2002 were included.

SEER is a comprehensive population-based database in the United States that includes disease stage at initial diagnosis, initial treatments performed, and accurate data regarding patient survival. However, NPC is a much more commonly diagnosed disease in Southeastern Asia as compared to United States. Nevertheless, SEER remains an ideal approach to studying the survival of patients diagnosed with mNPC especially in recent time periods due to the relative completeness of the data.

In the present analyses, we included a total of 679 patients diagnosed with mNPC at initial presentation from the 1988 to 2012 SEER database. These patients were treated in the “real-world” setting as compared with potentially selected patients included in a clinical trial or single institutional experience. We chose to include only mNPC patients diagnosed between 1988 and 2012, it can be assumed that chemotherapy was widely used for patients with metastatic NPC during the time period. We discovered that the use of radiation therapy, to the primary disease and/or the metastatic foci, significantly improved overall survival (OS) and cancer specific survival (CSS). These results were consistent with those of earlier unpublished SEER based analyses, case reports, as well as those of the retrospective studies. Our SEER data analysis of 679 patients provides additional evidence (level of evidence: 2b). As such, we consider the current recommendation from the NCCN guideline of radiation therapy in patients diagnosed with metastatic NPC after achieving CR to systemic chemotherapy is reasonable. Nevertheless, whether it is appropriate to omit radiotherapy in patients who achieved partial response or stable disease after chemotherapy cannot be supported by our findings.

Mechanisms underlying the survival benefit of the local radiation therapy to mNPC remain unknown. Eliminating the primary tumor burden of nasopharyngeal cancer, which is close to the critical organs such as carotid arteries, brain, and brainstem, and could reduce the probability of death by uncontrolled local disease progression. In addition, tumor burden reduction could reduce the primary or secondary source of cancer cells for metastasis by “self-seeding”^20^. Studies have also demonstrated that increased serum EBV-DNA counts, an indicator of disease burden, is related to disease progression and reduced survival^21^. Treatment of the primary or metastatic foci of NPC may therefore reduce the number of circulating tumor cells. Another potential mechanism in favor of radiation to local disease foci could be removing tumor-promoting factors and immunosuppressive cytokines.

Retrospective studies are inherited with pitfalls such as selection bias. For example, it is unclear whether patients received radiotherapy in this cohort were healthier or have more symptoms that need symptomatic control. Certain variables unavailable from SEER database limited our analysis and precluded controlling for any potential selection bias. These variables include site-specific codes for radiation therapy. Therefore, it is not possible to differentiate whether the survival benefits were derived from irradiating the primary or metastatic foci. This question would be best answered in a large-scale retrospective study or prospective trial. Another drawback is that the regimen/timing/dosage of chemotherapy and schedule of chemotherapy relative to radiation is unknown. The lack of chemotherapy details is especially important given the influence of chemotherapy on mNPC progression and survival. However, as in our matched cohort, radiation and non-radiation groups were matched and comparable in terms of all demographic and clinical characteristics. These characteristics were critical in determining the other variables including treatment modalities, thus the use of chemotherapy, which is considered the primary treatment for NPC, would be expected to be similar between the 2 groups. SEER database also lacks information regarding the extent of the metastatic foci, an entity that undoubtedly influences patient survival. In addition, we also have no data on patients’ performance status and comorbidity. Despite of these insufficiencies, it is clear to us that tumor burden reduction from local therapy significantly improved overall survival in NPC patients diagnosed with metastasis, although further investigations are needed to understand the optimal application of radiation therapy that can maximize such benefit.

To our knowledge this is the first published population based study to evaluate the effectiveness of local RT in patients with mNPC. However, we only addressed the patients presented with DM at initial diagnosis. For patients develop distant failure or both distant and local failure after definitive treatment for their originally localized disease, whether radiation therapy to the metastatic foci (or both metastatic and local recurrent foci) at the time or recurrence provide a survival benefit was not addressed in our study. Results from a number of studies have indicated that DM occurred after definitive treatment of NPC has better long-term survival as compared to those presented at initial diagnosis^22^. Therefore, local treatment might be more important in salvaging NPC patients who failed distantly after definitive treatment.

## Conclusion

Despite the inherent limitations of this SEER-based study, our results suggest that radiation therapy provided to NPC patients with distant metastasis at diagnosis confers a survival benefit. Because of the lack of site-specific EBRT codes, it was not possible to examine the difference of the survival advantage of radiation therapy to the primary or metastatic sites on patient survival. Nevertheless, our results, in combination with results from other retrospective series, suggest that the recommendation of radiation therapy for local disease foci in the head and neck region after sufficient disease control by chemotherapy from the NCCN guideline is reasonable. Nevertheless, large-scale retrospective studies or organized prospective clinical trials designed not only to demonstrate a survival advantage with radiation of the primary tumor but also to identify patients most likely to benefit is necessary.
